# Tears Falling on Goosebumps: Co-occurrence of Emotional Lacrimation and Emotional Piloerection Indicates a Psychophysiological Climax in Emotional Arousal

**DOI:** 10.3389/fpsyg.2017.00041

**Published:** 2017-02-07

**Authors:** Eugen Wassiliwizky, Thomas Jacobsen, Jan Heinrich, Manuel Schneiderbauer, Winfried Menninghaus

**Affiliations:** ^1^Language and Literature Department, Max Planck Institute for Empirical AestheticsFrankfurt am Main, Germany; ^2^Department of Education and Psychology, Freie Universität BerlinBerlin, Germany; ^3^Experimental Psychology Unit, Helmut Schmidt University/University of the Federal Armed Forces HamburgHamburg, Germany; ^4^Departments of Time-based Media and Film, University of Fine Arts HamburgHamburg, Germany

**Keywords:** emotional tears, piloerection, being moved, psychophysiology, film

## Abstract

This psychophysiological study is the first to examine the relationship between emotional tears and emotional piloerection (i.e., goosebumps). Although both phenomena have been related to peak states of being moved, details about their temporal occurrence and the associated levels of physiological arousal have remained unknown. In our study, we used emotionally powerful film scenes that were self-selected by participants. Our findings show that even within peak moments of emotional arousal, a gradation of intensity is possible. The overlap of tears and goosebumps signifies a maximal climax within peak moments. On the side of the stimulus, we found that displays of prosocial behavior play a crucial role in the elicitation of tears and goosebumps. Finally, based on the results of a formal film analysis of the tears-eliciting clips provided by our participants, as compared to randomly extracted, equally long control clips from the same films, we show how the technical and artistic making of the clips was optimized for the display of social interaction and emotional expressions.

It is such a secret place, the land of tears.

— Antoine de Saint-Exupéry,

The Little Prince

## Introduction

Being moved to tears by an artwork is certainly nothing one would easily forget. Although many people experience emotional episodes of this type from time to time, emotional lacrimation remains “a secret place” we know very little about. Previous research has distinguished five subgroups of emotional tears^1^ dependent on five types of elicitors (Vingerhoets, 2013; Denckla et al., 2014): (1) tears in response to physical pain, (2) tears related to personal loss, e.g., when a close person dies, (3) tears related to empathic pain, e.g., when a close person faces hard times, (4) societal tears, e.g., ritual weeping, and (5) art-elicited tears.^2^ Tears of the latter type is the only category that people do not try to avoid. Quite the contrary, a sad film that moves its audience to tears will be regarded as a good film and will be more likely to receive higher ratings on a scale that measures how much viewers want to see the film again (Hanich et al., 2014; Wassiliwizky et al., 2015). This peculiar phenomenon has been explained by the fact that being moved, although encompassing negative emotional ingredients such as sadness, is an overall pleasurable emotional state (Tokaji, 2003; Tan, 2009; Benedek and Kaernbach, 2011; Hanich et al., 2014; Menninghaus et al., 2015; Wassiliwizky et al., 2015). Importantly, sadness has to be balanced by a positive emotional antidote for a scenario to be of the *sadly moving* type (Wassiliwizky et al., 2015). In a farewell scenario, for instance, the predominant emotion of sadness is mixed with positive feelings of social bonding. Conversely, negative affect is routinely an emotional antidote in the case of *joyfully moving* scenarios. Here the predominant building block of joy—elicited, for instance, in a reunion scene—is balanced by a negative antidote, that is, reactivated feelings of the preceding painful separation of the characters. Thus, being moved is a typical mixed emotion (Cacioppo and Berntson, 1999). For empirical research, it is crucial to distinguish between sadness, which is an unpleasant emotional state that humans try to avoid, and being sadly moved—an emotional state for which people willingly spend money when they buy cinema, theater, and opera tickets, film and music recordings, books, and so forth (cf. Cova and Deonna, 2014; Brattico et al., 2016).

The linkage between feelings of being moved and tears in the cross-cultural expression “moved to tears” is more than a mere coincidence (“zu Tränen gerührt” in German, “tot tranen geroerd” in Dutch, “ému jusqu’ aux larmes” in French, “commosso fino alle lacrime” in Italian, “conmoviendo hasta las lágrimas” in Spanish, “rastrogan do slez” 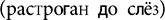 in Russian, “dojatý k slzám” in Czeck, “gǎn dòng de luò lèi” ([scale=.50]img001) in Chinese, “kanrui” ([scale=.50]img002) in Japanese). Several studies have shown that art-elicited tears can be regarded as physiological indicators of being moved (Scherer and Zentner, 2001; Scherer et al., 2002; Kuehnast et al., 2014). Two other physiological markers have also been shown to accompany feelings of being moved: emotional piloerection, that is, goosebumps (Benedek and Kaernbach, 2011; Wassiliwizky et al., 2015), and a lump in the throat (Scherer et al., 2002). Moreover, all these studies emphasize the salience of the physiological arousal (such as heart palpitations, heavy breathing, sweaty palms) that participants report when shedding art-elicited tears or experiencing goosebumps or a lump in the throat.

To date, however, research on the psychophysiological correlates of emotional tears in general and art-elicited tears in particular is utterly scarce (Kraemer and Hastrup, 1988; Gross et al., 1994). Moreover, although there is a good deal of literature on the psychophysiological correlates of emotional goosebumps and chills, which represent the subjective feeling component of piloerection episodes (among others Blood and Zatorre, 2001; Rickard, 2004; Grewe et al., 2007; Salimpoor et al., 2009, 2011; Benedek and Kaernbach, 2011), we do not know how emotional goosebumps interact with emotional tears. That is, do emotional tears and emotional piloerection overlap, or does one always precede the other? Is there a higher physiological arousal when the two responses overlap? Is it even possible for them to overlap, since they are governed by two antagonistic branches of the autonomous nervous system (ANS) (the sympathicus in the case of piloerection and the parasympathicus in the case of tears)? Moreover, according to a theory put forward by several researchers (Bindra, 1972; Efran and Spangler, 1979; Frijda, 1986), emotional tears initiate a recovery process after a period of peak arousal (which in our case would be indicated by goosebumps). Therefore, the recovery hypothesis would predict that tears should always come after emotional piloerection.

The aim of the present study was to systematically investigate the interrelation between tears and goosebumps, both temporally and in terms of their psychophysiological arousal signatures, including skin conductance, cardiovascular and respirational measures, and facial electromyographic activity. Most of the current theories on emotion agree on the fact that both physiological arousal of the ANS and facial expressions of emotions constitute two major components of an emotional episode (Ekman, 1993; Scherer, 2009; Kreibig, 2010). Two facial muscles—corrugator supercilii and zygomaticus major (cf. Supplementary Figure S1)—have repeatedly been demonstrated to indicate negative and positive affect, respectively (Cacioppo et al., 1986; Witvliet and Vrana, 1995; Lang et al., 1998; Larsen et al., 2003; Aue and Scherer, 2008; Lundqvist et al., 2008). The unintentional activations of these two muscles thus provide continuous measures of negative and positive affect. Importantly, collecting electromyographic data of these two facial muscles may even allow us to investigate mixed emotional states, if both muscles are activated in periods of tears and goosebumps. This would support former claims about the mixed nature of states of being moved (Tan, 2009; Benedek and Kaernbach, 2011; Hanich et al., 2014; Kuehnast et al., 2014; Menninghaus et al., 2015; Wassiliwizky et al., 2015).

Because emotional piloerection marks states of peak emotional arousal and heightened skin conductance is indicative for physiological arousal driven by sympathetic activity (Benedek and Kaernbach, 2010), one should expect increased skin conductance responses for these emotional episodes. This is exactly what was found in a substantial number of studies on piloerection and chills in response to music and film (Rickard, 2004; Grewe et al., 2007, 2009; Guhn et al., 2007; Salimpoor et al., 2009, 2011; Benedek and Kaernbach, 2011; Sumpf et al., 2015). Similarly, several studies have shown increased cardiovascular and respirational activity in piloerection/chills episodes (Blood and Zatorre, 2001; Grewe et al., 2009; Salimpoor et al., 2009; Benedek and Kaernbach, 2011). However, some studies have failed to replicate these latter effects (Rickard, 2004; Guhn et al., 2007); hence the findings for cardiovascular and respirational activity appear to be less consistent than those for skin conductance. At the same time, the few studies that investigated emotional lacrimation report both increased electrodermal activity, faster rates of heartbeats and breathing in lacrimation periods (Kraemer and Hastrup, 1988; Gross et al., 1994). These results question the validity of the recovery hypothesis (Bindra, 1972; Efran and Spangler, 1979), which predicts reduced sympathetic activity in lacrimation periods and thus lower heart rates, slower breathing, and decreased skin conductance. Our data allowed to test these contrasting predictions against each other.

To ensure maximal responses, we relied on stimuli that were self-selected by participants before the actual study. Specifically, we asked each participant to provide individual film excerpts that reliably move him or her to tears (for a similar approach see, e.g., Blood and Zatorre, 2001; Salimpoor et al., 2009, 2011). Moreover, we measured the piloerection objectively by means of a video recording device, the “goosecam” (Benedek et al., 2010; Benedek and Kaernbach, 2011; Sumpf et al., 2015), which we constructed for this investigation.

One of the most debated issues in research on emotional tears is their (evolutionary) adaptive function. The viewpoints range from a “purposeless, incidental result” (Darwin, 1872, p. 175) to more recent positions that highlight the social benefits of emotional tears (Gross et al., 1994; Vingerhoets, 2013). According to the latter view, people who shed emotional tears are more likely to be empathized with and to receive social support (Balsters et al., 2013; Vingerhoets et al., 2016). The importance of a social component on the side of the perceiver as well as in the stimulus has also repeatedly been highlighted in experimental studies on being moved and chills. Panksepp (1995), for instance, associated his findings on sad songs and the concomitant elicitation of chills with the sudden arousal of social emotions and feelings of social bonding in the listener; however, he did not investigate this issue systematically. Similarly, using emotionally moving film scenarios that elicited chills, Wassiliwizky et al. (2015) concluded that sadness accompanied by prosocial emotions is one of the most powerful stimuli for episodes of being moved to occur and to be accompanied by chills. The social component thus appears to play a crucial role in states of being moved.

We wanted to further elaborate on this issue by investigating features of the tears-eliciting clips provided by our participants. Specifically, we expected a high percentage of the clips to display social interactions. We planned to examine the predominant group size of the characters in the narrative foreground as well as the number of onlookers or bystanders. By virtue of being in a witnessing position, this latter group of characters is comparable to that of an actual film viewer, and hence may serve to prepare, prime, and facilitate the responses of the actual film viewer (Hanich et al., 2014; Hanich and Menninghaus, in press). Moreover, we planned to investigate several technical aspects of the clips, such as the camera distance, lighting, and camera perspective. Given our hypotheses regarding the importance of social interactions, we hypothesized that the technical aspects of the scenes would optimize displays of protagonists’ and bystanders’ facial expressions, that is, there would be a predominance of close-ups and eye-level perspectives. Finally, we wanted to identify the dominant film genres in the stimulus pool.

## Experiment

### Participants

Twenty-five right-handed German native speakers (3 men, *M* = 28.8 years, *SD* = 8.5), all of them university students of different disciplines, were tested. All participants reported having normal vision and hearing. Prior to the study, the participants gave their written consent. They were compensated with 25 EUR at the end of the study.

### Stimuli

Several weeks before the testing, each participant provided a set of three to six emotionally powerful film scenes which contained peaking moments of emotional arousal and had repeatedly moved him or her to tears in the past. This resulted in a total stimulus pool of 137 different clips (several identical excerpts were chosen by multiple participants). The average clip length was 249 s; the lengths ranged between 53 and 666 s. Most of the films from which the excerpts were taken were produced in the US (80.28%), followed by the UK (16.90%), Germany (14.08%), and France (9.86%; note that because of co-productions such as Germany/France these percentages do not add up to 100). The production years ranged from 1941 (*Citizen Kane*) to 2012 (*Les Misérables*). For a complete list of the films, see Supplementary Table S7. All clips from non-German films were shown in the dubbed German version. We did not manipulate any parameters of the clips except for adding two-second black fade-ins and fade-outs to smooth the transitions at the beginning and end of each clip. During the testing, all participants watched only their self-selected clips.

### Procedure

Participants completed the experiment while sitting comfortably in a reclining armchair, with their non-dominant forearm placed on an armrest. With lights dimmed and all distractions minimized, the lab was turned into a comfortable and relaxed environment. To ensure a private atmosphere, the experimenter left the testing room after providing instructions and attaching the physiological sensors. The testing began with an initial baseline of 5 min, followed by the self-selected film clips in randomized order. Presentation^®^14.9 (Neurobehavioral Systems, San Francisco, CA, USA) was used to present the stimuli. The sound was played binaurally through high-quality K618-DJ headphones (AKG Acoustics, Vienna, Austria). Participants were asked to monitor their emotional responses and to push a button with their dominant hand when they felt moved to tears (for the entire duration of the experience). After each clip, they were requested to rate the intensity of their emotional lacrimation on a 6-point Likert scale. The levels of the scale were verbally anchored as follows: 0 = *no emotional arousal*; 1 = *slight emotional arousal*; 2 = *slight feeling of tears*; 3 = *strong feeling of tears*; 4 = *eyes filled with tears*; 5 = *tears rolling down the cheeks*. A relaxation pause of 1 min followed the rating to prevent potential carry-over effects. On top of collecting the standardized ratings of the intensity of the participants’ tear responses after each clip, we explored the subjective feeling component of their tear episodes by asking them for verbal statements during the debriefing after the testing session. Specifically, we asked them to describe how it felt to be moved to tears and whether they experienced this as rather pleasant or unpleasant. (Notably, these latter data were not collected for purposes of a rigid formal analysis—which would require a much larger sample size (cf. Kuehnast et al., 2014)—but rather in an exploratory fashion and with a view to potentially inspire future research on this mostly neglected emotional component). The entire testing took roughly 45 min per participant.

### Measurement and Preprocessing

Psychophysiological data were acquired (Supplementary Figure S1) via a 10-channel bioamplifier, the Nexus-10, which included the recording software Biotrace (Mind Media B.V., Netherlands). To collect skin conductance data, two flat 10 mm Ag/AgCl dry electrodes were fixed at the phalanges of the ring and middle fingers of the non-dominant hand; the sampling rate was 32 Hz. A photoplethysmograph sensor placed on the phalanx of the index finger of the same hand digitally recorded blood volume pulse, sampled at 128 Hz. A belt containing stretch-sensitive sensors was placed around the participants’ diaphragms to assess respiratory activity; these data were sampled at 32 Hz. Facial electromyographic (EMG) activity was recorded at a sampling rate of 2048 Hz at the corrugator supercilii and zygomaticus major, in accordance with common recommendations for electrode placements (Fridlund and Cacioppo, 1986). The electrodes were prepared with an isotonic paste (CV-Tronic, Versmold, Germany). Continuous objective measurement of piloerection was carried out by means of a video recording device (the “goosecam”) that was constructed according to the instructions of Benedek et al. (2010). Because a recent study had found the legs to be the most likely place for piloerection to occur (Wassiliwizky et al., 2015), participants were asked to roll up one trouser leg, and the device was attached to the lower leg above the right calf muscle (Supplementary Figure S1). Further they were asked to keep the leg still during the presentation of a clip in order to prevent movement artifacts. The goosecam had a coverage of 3 cm × 5 cm of the skin surface. The recorded video data were analyzed oﬄine using the Matlab analysis software Gooselab V1.21^3^, which is based on a two-dimensional discrete Fourier transform for every frame of the video (Supplementary Figure S2). The resulting continuous measure of piloerection was compared to the values of the baseline in order to obtain the exact onsets and offsets of piloerection incidents (Benedek et al., 2010; Benedek and Kaernbach, 2011). Additionally, all video recordings were checked in a control inspection using VirtualDub 1.10.4^4^ in order to confirm the outcomes of the Gooselab analysis and to exclude potential artifacts (no critical artifacts were detected).

Skin conductance data were analyzed using the Matlab analysis software Ledalab V3.4.4^5^. We used Continuous Decomposition Analysis (Benedek and Kaernbach, 2010), which disentangles and extracts single skin conductance responses (SCRs) in a way that ensures that the amplitude of a later response is not influenced by the proximity of the preceding response activity. For our statistical analysis, we used the phasic electrodermal activity (pEDA) component (Benedek and Kaernbach, 2010), which reflects the rapidly varying (over seconds) activity of skin conductance responses, in contrast to the slowly varying (over minutes) tonic activity (i.e., the skin conductance level). In a former study, the tonic activity did not show any significant changes in piloerection phases (Benedek and Kaernbach, 2011). The heart rate was derived from the raw blood volume pulse data by means of a peak detection algorithm integrated in the Biotrace software (sampled at 32 Hz). In a similar way, the respiratory rate was computed based on the signal recorded through the respiration belt, resulting in a continuous measure sampled at 32 Hz. The EMG signal was band-pass filtered within the frequency range of 20–500 Hz, which is the predominant range of facial EMG signals (Fridlund and Cacioppo, 1986), and convolved with a fourth-order Butterworth low-pass filter. From this preprocessed data, root mean square amplitudes were computed for the corrugator and the zygomaticus activity at a sampling rate of 32 Hz. Afterwards, all psychophysiological data were adjusted to account for inter-individual baseline differences among the participants by subtracting the individual baseline score (averaged over 5 min) from the respective signal (resulting in reactivity scores) and dividing the difference scores by the individual standard deviation of the baseline. The resulting normalized (i.e., linearly z-transformed) scores reflect changes from the initial baseline period due to experimental stimulation (for similar normalization procedures see, e.g., Salimpoor et al., 2009; Benedek and Kaernbach, 2011). In a final step, all physiological data were down-sampled to 2 Hz before entering the statistical analysis.

### Statistical Analysis

The statistical analyses were conducted using R 3.2.1 (R Development Core Team, 2015). To account for the hierarchical structure of the physiological data, with stimuli at level 1 and participants at level 2, we used mixed-effect analyses of variances. For each of the physiological measures, we conducted a 2 × 2 mixed-effect ANOVA with two binary factors: “Tears” (coding the occurrence of tears as indicated by button presses) and “Goosebumps” (coding the occurrence of emotional piloerection as captured by the goosecam and analyzed with Gooselab). The statistical model allowed for interaction effects, because we were interested in physiological correlates of the time periods in which tears overlapped with goosebumps. Exposure time spans in which neither tears nor goosebumps were observed were regarded as control time. Thus, all data points of the entire duration of the clips were considered for the statistical analysis. After the ANOVAs, pairwise Tukey *post hoc* tests (at a Bonferroni corrected significance level of α = 0.05) were conducted using the least-squares means.

### Formal Film Analysis

In order to identify formal filmic characteristics that prevail in tears-eliciting clips we had to compare them with an appropriate control set. To this end, we extracted control clips of the same lengths as the tears-eliciting clips from the same movies. For instance, if a 5 min clip from *Titanic* was used in the study to elicit tears in a certain participant, we extracted a control clip of 5 min from the same movie starting at a random time position. The random time position was generated using R; the extraction began from the next shot change that followed the random time point. If the control clip overlapped with the tears-eliciting clip, another random position was generated. Importantly, this approach keeps a great number of technical, aesthetic, and production related parameters constant between the tears eliciting clips and their matched control clips, thereby allowing for formal statistical testing of which features differ between both sets^6^.

In coding the formal filmic characteristics of both sets, we relied on a standard formal film analysis system by Bordwell and Thomson (2013). First, we focused on the camera’s perspective, the camera distance (also called framing), and the lighting. Both the camera’s perspective and the distance are described in terms of their relation to the depicted character. The distance or framing refers to the scale in which a particular character, or only parts of this character, is shown in the image. In line with Bordwell and Thomson (2013), we considered seven camera distances: close-up (depicts details of the character, most commonly the face), extreme close-up (singles out a portion of the face or magnifies an object), medium close-up (frames the character from chest up), medium shot (frames the character from waist up), medium-long shot (frames from the knees up), long shot (depicts characters in full length), and extreme long shot (depicts landscapes, the human figures are tiny or lost). The perspective refers to the angle at which the camera is positioned in relation to the character and the character’s eyes. We used five different perspectives: eye-level view (also called straight-on angle in which the camera is positioned orthogonally in respect to the object; usually, the camera is at the level of the character’s eyes), low-angle view (the camera is looking up), high-angle view (the camera is looking down), worm’s view (extreme low-angle view), and bird’s view (extreme high-angle view). We also coded point-of-view shots (POV), in which the camera creates an illusion that the film viewer sees through the eyes of the character. With respect to the lighting, we distinguished between high-key lighting (soft lighting, low in contrast, detailed in shadow areas) and low-key lighting (hard lighting, strong contrast, dark shadows, low/no fill light). We also examined the importance of music in the clips: if music was present, we distinguished between music as a musical score outside of the film story world (i.e., non-diegetic), non-diegetic singing with instruments, non-diegetic singing without instruments, depiction of music making within the film story world, and depiction of singing within the film story world. Finally, we coded for the depiction of crying characters. Given the fact that emotional expressions can be contagious for the viewer (Hatfield et al., 1994) and that film makers use this phenomenon in order to facilitate emotional arousal in the viewer, we expected to find more crying characters in the tears-eliciting clips than in the randomly chosen control clips. All parameters were coded for each shot of both the experimental (i.e., tears-eliciting) and the randomly chosen, equally long control clips. Because this procedure is extremely time demanding, we restricted the coding to 50 random experimental and 50 corresponding control clips. The coding was performed by an external professional coder (a film scholar), who was not familiar with the details of the analysis.

In addition to the formal filmic characteristics of the clips, we also analyzed the social dimension of the scenarios. First, we identified the clips that depict social interactions (from the complete experimental set of 137 clips); here we differentiated between human interactions, human–animal (and animal–animal) interactions, and interactions with anthropomorphic characters. We further examined the subset with human interactions by categorizing the number of interacting characters (one, two, three, four, or five and more). The same analysis was conducted for bystanders or witnesses, who are typically shown in the background of a scene.

## Results

### Occurrence of Tears and Goosebumps

All participants indicated that they experienced emotional tears. A total of 308 incidences was reported, translating into 0.55 incidence per minute (*M* = 29.06 s, *SD* = 1.94, range 0.5–325.25 s). The goosecam video recordings of 10 participants (40% of the sample) showed objective piloerection, 75 incidences in total, or 0.32 incidence/min (*M* = 33.81 s, *SD* = 21.18, range 5–121 s). The percentage of the sample showing positive evidence of piloerection is consistent with previous studies that used a variety of other stimuli, such as films, music (40%; Sumpf et al., 2015) and audio film soundtracks (43.1%; Benedek and Kaernbach, 2011). Notably, piloerection appeared primarily (80%) in response to clips that triggered a full-blown lacrimation response (levels 4 and 5 of the tears scale, i.e., eyes filled with tears and tears rolling down the cheeks) rather than a mere “feeling like crying” (levels 2 and 3 on the tears scale).

### Sequence Effects

Of the goosebumps periods, that is, periods during which objective piloerection can be observed in the video recording, 58.7% overlapped with tear periods, that is, periods when the response button was pushed (the overlap time of tears and goosebumps was on average 22.43 s, *SD* = 19.7, min = 1.75, max = 100.72). In 59.1% of these cases, the tears response preceded the piloerection, while in the remaining 40.9%, the goosebumps came first. The Pearson’s chi-square test showed no difference between these two distributions (χ^2^ = 1.45; *df* = 1; *p* = 0.228). Thus, our study observed no apparent temporal order effects for goosebumps and tears.

### Psychophysiological Correlates

**FIGURE 1:**
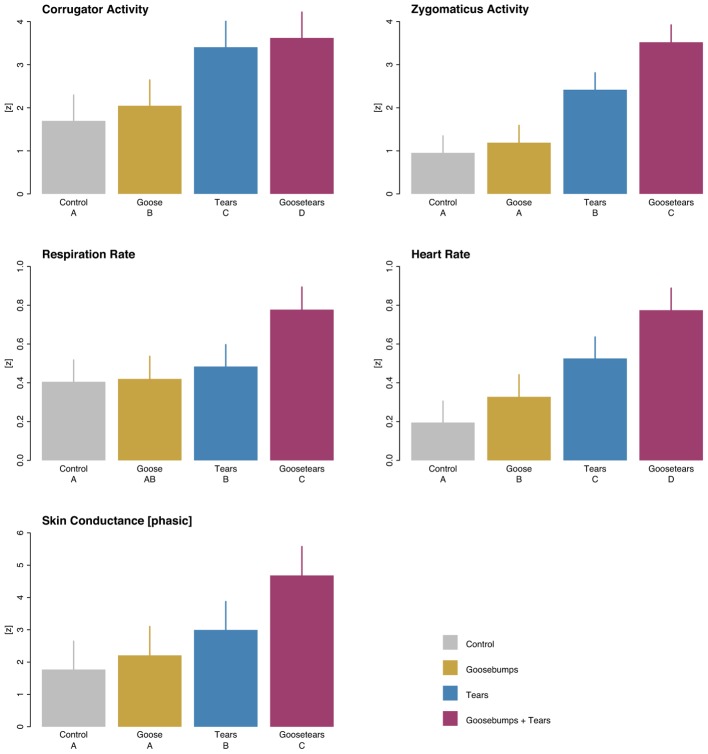
**Psychophysiological correlates in response to tears-eliciting film clips.** A gradual increase of psychophysiological arousal was observed, beginning with emotional piloerection (captured objectively by the goosecam), followed by emotional tears (self-indicated by participants) and then climaxing in the overlap of both responses (control refers to stimulus time stretches in which neither of the two responses was observed). Error bars indicate standard errors as estimated in multilevel mixed-effect models. Bars with the same letter are not significantly different from each other at the 0.05 level. (Please note that for ease of readability, the scaling differs between rows).

**FIGURE 2:**
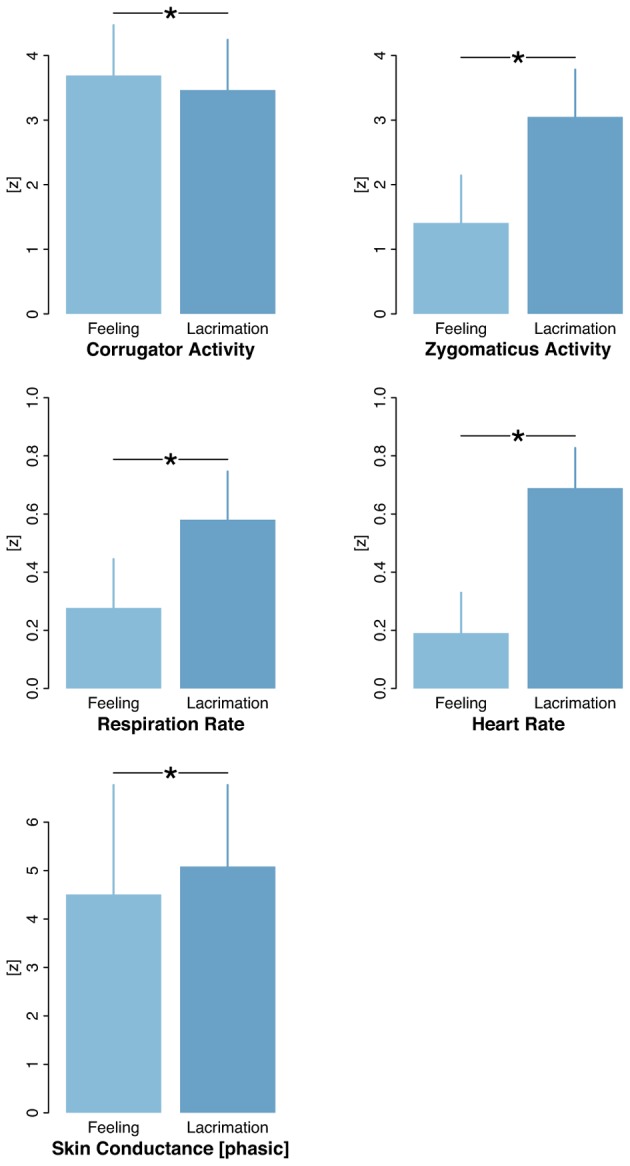
**Psychophysiological correlates of a second analysis in which the “feeling like crying” was compared with the actual shedding of tears.** In general, the full-blown lacrimation triggered significantly higher arousal states (at the 0.05 level) than the mere feeling like crying. Error bars indicate standard errors as estimated in multilevel mixed-effect models. (Please note that for ease of readability, the scaling differs between rows).

The psychophysiological correlates of the autonomous nervous system and of the electromyographic facial activity revealed a stable result pattern across different domains. In general, emotional piloerection showed greater amplitudes compared to control time, followed by amplitudes for emotional tears and climaxing eventually in periods during which tears overlapped with goosebumps (**Figure 1**; for the underlying statistics see Supplementary Table S1). Moreover, within the tears incidences, periods that were rated 4 and 5 on the tears scale, that is, full-blown lacrimation, triggered higher psychophysiological responses than periods that were rated with 2 and 3 on the tears scale, that is, only a feeling like crying (**Figure 2**; for the underlying statistics see Supplementary Table S2).

Because the electromyographic activity of the corrugator (which is associated with negative affect) and the zygomaticus (which is associated with positive affect) revealed a similar result pattern, we further examined the co-occurrence of these two activities within clips, and more specifically within episodes of tears as well as within episodes of goosebumps. For this purpose, we performed a correlational analysis for the measures of both muscles after they were averaged per participant for the entire clip (for the first analysis), for the tear episodes (for the second analysis), and for the goosebumps episodes (for the third analysis). Pearson’s product-moment correlation of the corrugator and zygomaticus activities for the entire clips amounted to *r* = 0.20 (*t*_130_ = 2.38, *p* = 0.0187); for the tear episodes, *r* = 0.24 (*t*_109_ = 2.62, *p* = 0.0099); and for the goosebumps episodes, *r* = 0.21 (*t*_28_ = 1.17, *p* = 0.2535). Thus, the analyses show a moderate but significant co-activation of both muscles at the level of the entire clips and at the level of tear episodes.

### Formal Film Analysis

**FIGURE 3:**
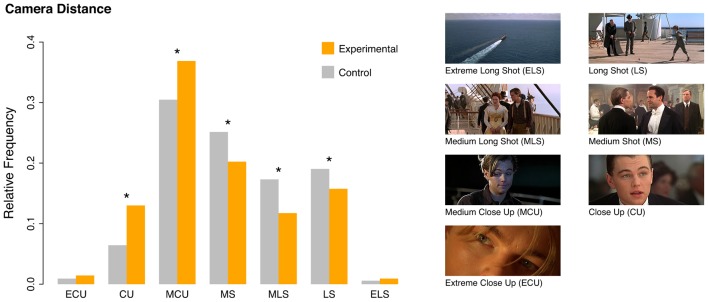
**Comparison of camera distances between tears-eliciting clips and a matched control set.** The ordinate shows the relative frequency accumulated over the duration of all clips in a respective stimulus set. In general, tears-eliciting clips tended to draw predominantly on camera positions close to the character (i.e., significantly more close-ups and medium close-ups), whereas for the control set significantly more longer camera distances (i.e., medium shot, medium long shot, long shot) were found (for details, see Supplementary Table S3). The pictures illustrating different camera distances were taken from Titanic (1997).

In the first step, we identified the prevalent genres that the clips were taken from (according to the classification of the Internet Movie Data Base, IMDb)^7^. Supplementary Figure S3 shows that Drama was the most common genre (38.60%), followed by Romance (18.54%), Comedy (7.90%), and Biography (5.17%). All remaining genre types figured below 5%. This outcome fits well with the scenario types, which primarily focused on saddening content (67.15% of the clips) such as death bed scenarios, break-ups, and farewell scenarios. In contrast, 32.85% of the clips focused on joyful events, such as reunions and the achievement of important goals (for a detailed list of scenario types, see Supplementary Table S7). In the technical part of the formal film analysis, that compared the experimental tears-eliciting clips with matched control clips, we focused primarily on camera distance and camera perspective. **Figure 3** shows profiles of camera distance usages for both sets, differing significantly from each other in a Pearson’s chi square test (χ^2^ = 126.64; *df* = 6; *p* < 0.001). Although both the tears-eliciting and the control clips tended to draw mostly on medium close-ups, this preference was significantly higher for the tears-eliciting clips: Whereas roughly 37% of the total time of the analyzed 50 tears-eliciting clips were shot in a medium close-up, the matched control set used this framing only in about 30% of the total time of all 50 clips (for statistical details, see Supplementary Table S3). Similarly, tears-eliciting clips used significantly more close-ups, whereas the matched controls showed significantly more medium shots, medium-long shots, and long shots.

**FIGURE 4:**
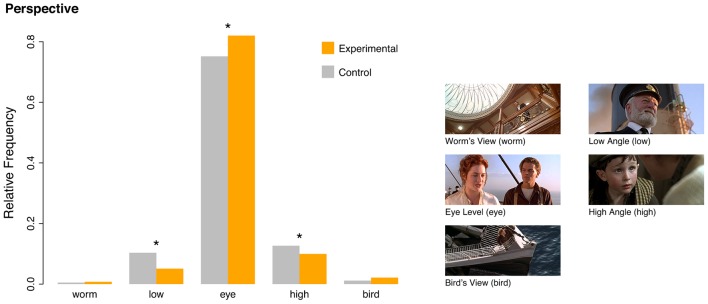
**Comparison of camera perspectives between tears-eliciting clips and a matched control set.** Tears-eliciting clips used significantly more eye-level/straight-on views, whereas for the control set significantly more high and low angle perspectives were found (for details, see Supplementary Table S4). The pictures illustrating different perspectives were taken from Titanic (1997).

Regarding the perspective, the eye level (or straight-on angle) was predominant for both sets (**Figure 4**). Tears-eliciting clips, however, used this perspective significantly more than their matched controls, which in turn showed more low and high angle perspectives (the distributions differed significantly from each other in a Pearson’s chi square test: *χ^2^* = 68.08; *df* = 4; *p* < 0.001; see also Supplementary Table S4). For the lighting, we found a general predominance of high key lighting (Supplementary Figure S4). Tears-eliciting clips, however, used significantly less high key and consequently more low key lighting (χ^2^ = 61.73; *df* = 1; *p* < 0.001; see also Supplementary Table S5). Although point-of-view shots are generally rare (Supplementary Figure S4 and Table S5), tears-eliciting clips used slightly more of them than the control set (*χ^2^* = 14.43; *df* = 1; *p* < 0.001). Similarly, clips that elicited tears in the participants, depicted significantly more crying characters than randomly chosen control clips (*χ^2^* = 183.97; *df* = 1; *p* < 0.001; see also Supplementary Figure S4 and Supplementary Table S5). For the usage of music, we observed a greater tendency for tears-eliciting clips both to underlie scenarios with music and songs from outside of the story world and to depict singing characters within the story world (*χ^2^* = 251.43; *df* = 5; *p* < 0.001; see also Supplementary Figure S4 and Supplementary Table S5).

**FIGURE 5:**
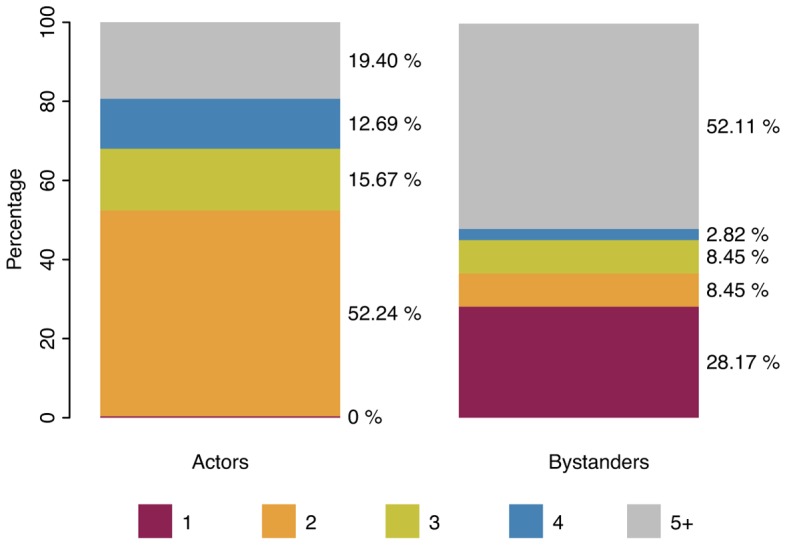
**Percentages of tears-eliciting clips displaying different numbers of actors and bystanders in the foreground and background of the scenario.** Note that whereas dyads tended to dominate for the main characters in the foreground, single witnesses and groups of five or more were particularly frequent for the bystanders in the background. The difference was significant in a Pearson’s chi-squared test (χ^2^ = 83.12; *df* = 4; *p* < 0.001).

As we expected, the majority of the experimental clips, 93.66%, displayed social interactions: 69.72% displayed human interactions, 12.68% human-animal or animal-animal interactions, and 11.27% interactions with anthropomorphic characters (e.g., E.T., the extra-terrestrial); only 6.34% displayed no interactions at all (i.e., a single character was depicted). We further examined the number of interacting characters as well as the number of bystanders/witnesses (45% of the clips displayed only main characters, with no bystanders in the narrative background). The results are shown as stacked bar plots in **Figure 5**. Regarding the number of interacting characters, we observed a strong tendency toward dyads (52.24%), followed by groups with five or more characters interacting with each other (19.40%). Three and four persons were each displayed in about 15% of the clips. Regarding the bystanders, we observed a diverging pattern. Here, single witnesses (28.17%) and groups of five up to large crowds (52.11%) were the most common types. The differences in the group sizes for actors and bystanders were significant in a Pearson’s chi-squared test (*χ^2^* = 83.12; *df* = 4; *p* < 0.001).

## Discussion

It has been claimed that emotional tears are a uniquely human phenomenon (Frey, 1985; Provine, 2012; Trimble, 2012; Vingerhoets, 2013). Typically, humans weep in response to unpleasant or painful stimuli and unfortunate events. However, shedding tears in contexts of exposure to artworks and media products (which readily include elicitation of negative emotions) has been associated with very intense moments of the pleasurable emotional state of being moved (Scherer and Zentner, 2001; Scherer et al., 2002; Hanich et al., 2014; Kuehnast et al., 2014). Likewise, emotional piloerection has been demonstrated to correlate with the peaks of states of being moved (Benedek and Kaernbach, 2011; Wassiliwizky et al., 2015). The first aim of our study was to examine the psychophysiological correlates of these two indicators along with their interrelation. The results show a gradual effect in the intensity of psychophysiological arousal: the intensity was highest for tears combined with goosebumps, followed by tears only and goosebumps only^8^. This gradual effect was observed in all psychophysiological channels that were measured (**Figure 1**). As emotional empathy seems to play a crucial role in states of being moved (as discussed in more detail below), the present findings of heightened physiological arousal can be interpreted as lending support to the consistent reports of physiological activation when witnessing and empathizing with others in need (e.g., Sze et al., 2012).

To date, psychological and neuroscientific studies have reported the occurrence of goosebumps or chills during moments of peak emotional arousal in different aesthetic contexts (for music, see Salimpoor et al., 2011; for films, see Wassiliwizky et al., 2015). A few studies have also reported emotional tears during peak moments of art reception (Scherer and Zentner, 2001; Scherer et al., 2002; Hanich et al., 2014). Our data present evidence for a gradual increase in emotional arousal even within peak emotional moments, with overlapping tears and goosebumps representing the climax of arousal. The close relatedness of tears and goosebumps became obvious in the co-occurrence pattern both at the level of individual periods (almost 60% of the goosebump periods overlapped with tears) and at the level of clips (goosebumps appeared primarily, in 80% of the cases, in clips that triggered a full-blown lacrimation, in contrast to “feeling like crying”). The distinction between an actual shedding of tears and a mere awareness of an upcoming period of crying, i.e., without production of tears (cf. Pelowski, 2015), was corroborated by psychophysiological data that showed higher arousal states for the former category (except for the corrugator activity, **Figure 2**; the reverse effect for the corrugator activity can be interpreted as higher tension of facial muscles, which indicates negative affect before the emotional release happens, i.e., the lacrimation).

The overlap and close alliance of piloerection and lacrimation involves a complex underlying physiological orchestration. After all, these responses are governed by two antagonistic divisions of the ANS, which is generally concerned with regulation of fundamental bodily functions related to the activation of organs and tissues. Whereas the pilomotor reflex of the arrector pili muscles (around the hair follicles), which causes body hair to erect, is controlled exclusively by the sympathetic nervous system (SNS; Hellmann, 1963), the release of the lacrimal secretion from the lacrimal glands, in contrast, is modulated by the parasympathetic nervous system (PNS; Werb, 1983; Dart, 2009). The core functions of these two divisions of the ANS have classically been associated with mobilization of energy in demanding situations (the SNS) and regeneration in the absence of environmental stressors (the PNS). This has led researchers to assume that emotional lacrimation should bring about recovery and suppression of sympathetic activity (Efran and Spangler, 1979). Our present findings, however, point in the opposite direction (see also Kraemer and Hastrup, 1988; Gross et al., 1994), since we observed increased sympathetic activity (as reflected by the skin conductance and heart rate data) in periods of tears and no apparent effect of order (with tears always following goosebumps). Our results show that emotional tears, although governed by parasympathetic activation, can be accompanied by maximal sympathetic arousal when they overlap with emotional goosebumps.

Although most organs and glands are innervated by both the SNS and the PNS (Pape et al., 2014) in order to either activate or relax the respective effector, the antagonistic activations tend to alternate in time. In some complex physiological processes, however—for example, in sexual arousal, the two systems are known to interact simultaneously within the same process (Steger and Weidner, 2011, p. 67). We assume that intense states of being moved that are indicated by tears and goosebumps are likewise mediated by a complex antagonistic interplay of the two subdivisions of the ANS resulting in an intense physiological activation and a unique subjective bodily feeling. The cross-cultural expression of *feeling/being moved* is likely to reflect or at least be strongly informed by the experience of this heightened bodily arousal (cf. Kuehnast et al., 2014; Menninghaus et al., 2015). Moreover, PNS activation has also been associated with states of helplessness and feelings of being emotionally overwhelmed (Vingerhoets and Bylsma, 2015), which are typically reported by people who cry in response to movies (Denckla et al., 2014). Moreover, our participants reported immediately after the testing session that they felt greatly relieved after a period of tears (i.e., a cathartic effect; cf. Frey, 1985; Frijda, 1986) and that they enjoyed this experience, although they also reported that negative emotional components were involved (see Supplementary Table S6 for the original wording of the statements given by our participants after the testing session).

The mixed affective nature of the feelings of being moved was also reflected by moderate co-activation effects of the facial corrugator and zygomaticus muscles during periods of tears (and more globally, at the level of the entire clips)^9^. As stated above and elsewhere (Tokaji, 2003; Tan, 2009; Menninghaus et al., 2015; Wassiliwizky et al., 2015), the mixture of a predominant emotional component, for example, sadness, with its emotional antidote is an essential requirement for states of being moved to occur. Frequently, the antidote entails elements of high personal norms, moral goodness, and displays of prosocial behavior that lives up to high social norms and self-ideals. The significance of a prosocial dimension in the tears-eliciting stimuli was corroborated by our formal film analysis, as we will now explain.

First, most of our participants’ excerpts were taken from the genres of drama and romance, and hence from two genres that frequently address societal values and virtues such as altruism, bonding, self-sacrifice, faithfulness, and so forth. Moreover, almost all excerpts (about 94%) displayed social interactions involving humans, animals, or anthropomorphic characters. Second, as hypothesized, the formal filmic features of the individual clips were optimized for displaying the facial expressions of the protagonists and bystanders; this was reflected in a predominance of close-ups and an eye-level perspective as compared to the matched control clips. The function of an overall predominance of high key lighting (over 80% of total time for both stimulus sets) is to brighten the visibility of details like facial emotional cues (which might be the main reason for the prevalence of high-key lighting in dramas in general, as noted by Bordwell and Thomson, 2013, p. 129). The slight tendency of tears-eliciting clips towards more low-key lighting can be interpreted as further highlighting their gloomy content (cf. the Scenario column in Supplementary Table S7). Moreover, the experimental clips depicted significantly more crying individuals; this adds to emotional intensity by virtue of the tear effect (Provine et al., 2009; as discussed in more detail below), and by virtue of emotional contagion (Hatfield et al., 1994). Emotional intensity is further amplified by the use of musical scores and the depiction of singing characters. Music and singing have often been shown to be capable of eliciting peak emotional responses including piloerection/chills and tears on their own (Panksepp, 1995; Blood and Zatorre, 2001; Scherer and Zentner, 2001; Scherer et al., 2002; Rickard, 2004; Grewe et al., 2007, 2009; Guhn et al., 2007; Salimpoor et al., 2009, 2011; Fukui and Tyoshima, 2014). Finally, tears-eliciting clips used significantly more point-of-view shots which facilitate perspective taking and thereby empathic responses on the side of the film viewer.

Third, in 55% of all experimental clips picturing human interactions, we observed the presence of bystanders or witnesses in the narrative background of the scenario, such as a family member (e.g., an aunt who witnesses doctors telling the parents of a child who was seriously injured in an accident that their child has died), or a large crowd of strangers who witness the reunion of a couple (the main characters) after many years of separation. In general, we observed a strong tendency toward the display of either a single bystander or larger groups of five or more bystanders in the background of the scenarios. This stands in stark contrast to the general tendency of showing two actors in the narrative foreground of the scenarios. We suggest that this contrast results from the different dramatic functions that the characters fulfill in a scenario. As mentioned above, film makers often use bystanders to prime a particular response (e.g., crying) in the film viewer because the bystanders and film viewers are in similar witness positions. In the case of a single bystander, a considerable number of detailed cues can be displayed that will not escape the film viewer’s attention (a sad facial expression, trembling of the chin, sobbing, eyes hidden behind a hand, tears rolling down the cheek). For a large crowd, this level of detail is impossible; therefore, only simple, single cues are displayed by members of the crowd, such as cheering with arms raised. However, the sheer fact of a synchronized response in a large group of people makes for a powerful emotional stimulus. The prevalence of dyads in the narrative foreground, in contrast, might be due to the fact that most moving scenarios touch on issues that typically arise in very close personal and intimate relationships (e.g., romantic break-ups, declarations of love, farewells, marriage proposals, death bed scenarios; cf. the Scenario column in Supplementary Table S7).

The results of our study add to the converging evidence that states of being moved, the peaks of which involve emotional piloerection and/or emotional tears, draw substantially on the emotional resources of social cognition and social norms (cf. Vingerhoets and Bylsma, 2015). Interestingly, recent experimental studies have shown that participants who have been moved by an artwork or media product are more likely to display prosocial behavior in a subsequent behavioral test. Specifically, Stel et al. (2008) demonstrated that participants showed significantly greater readiness to make generous charitable donations if they were emotionally moved beforehand (for a similar line of research see Sze et al., 2012). Likewise, in a dictator game paradigm, Fukui and Tyoshima (2014) found enhanced empathy and altruism in participants who experienced music-elicited chills before playing the dictator game. Panksepp and Bernatzky (2002) offered an evolutionary explanation for the nexus between emotional piloerection and social cognition: They theorized that chills (which are readily associated with feeling cold) might have originated as a way to urge the organism to seek close social (and bodily) contact with others and thereby re-establish social bonds (see also Benedek and Kaernbach, 2011; Maruskin et al., 2012). Piloerection in response to artworks and media products could hence represent a derivative relic of this phylogenetically ancient response that still retains its function of fostering social bonds and facilitating prosocial behavior. Moreover, the fact that it is highly rewarding for an empathizing viewer to observe altruistic behavior in response to the plight and suffering of others indicates a potential evolutionary function of states of being moved for the reinforcement of group cohesion.^10^

Whereas piloerection urges the organism itself to re-establish social bonds (according to Panksepp’s theory, outlined above), the display of emotional tears has a complementary effect of attracting the social support of others for the crying individual. As mentioned earlier, the communicative signal of emotional tears has repeatedly been shown to compel others to provide help or emotional support to the crying individual (Hendriks et al., 2008; Vingerhoets et al., 2016). This may result from the fact that visible tears are attributed to greater amounts of emotional suffering. This *tear effect* has been corroborated by a number of studies that manipulated facial images by digitally adding (or removing) tears (Provine et al., 2009; Balsters et al., 2013; Vingerhoets et al., 2016). Moreover, visible tears have been shown to increase the perceived helplessness of the crier and a stronger willingness in the observer to provide help (Vingerhoets et al., 2016). As in the case of art-elicited piloerection, art-elicited tears might also represent an evolutionary relic that still serves the function of fostering social bonds between viewers and facilitates imitation of altruistic behavior (Stel et al., 2008).

We conclude that the elicitation of feelings of being moved could have a therapeutic value for people with low empathic capacities as well as in educational contexts. Classic cultural ideals as propagated by Schiller, Goethe, Lessing, and many other humanists typically include these transformative effects on the personality of readers, viewers, and listeners as *the* core function of emotionally moving arts altogether (e.g., Schiller, 2004; for more recent versions of this line of thinking see Nussbaum, 1997 and Oatley, 2016; for empirical evidence, see Kidd and Castano, 2013). Future research will have to assess the effectiveness of artworks and media products that are deeply moving in enhancing a person’s long-term capacity for empathy and the cognitive ability to adopt another person’s perspective.

## Limitations

Although we explicitly endorsed the participation of men in our study, only three out of the 25 participants were male. One could therefore question the generalizability of our results. However, a general bias toward the female gender is a well-established, cross-cultural finding in the scientific literature on emotional tears and crying (Bindra, 1972; Lombardo et al., 1983; Becht et al., 2001; for a review, see Vingerhoets and Scheirs, 2000). Females cry two to four times more frequently than males, starting in childhood (Jellesma and Vingerhoets, 2012) and continuing throughout their lives (Lombardo et al., 2001); moreover, critically for this study, women report higher rates of crying in response to sad films or sad books (Fischer et al., 2004) and also report enjoying these tears more than men do (Van der Bolt and Tellegen, 1996; Vingerhoets, 2013). Moreover, male crying is considerably less acceptable in most societies (MacArthur and Shields, 2015) and males feel more confused and irritated when confronted with other individuals—particularly other males—who start crying in their presence (Plas and Hoover Dempsey, 1988; Jesser, 1989).^11^ All these factors might have contributed to the bias of our sample toward females.

Since the presence of tears was not monitored objectively in our study and we relied solely on self-reports one could raise concerns regarding the demand characteristics, i.e., participants pushing the response button without actually experiencing tears. Although we initially planned to video record the faces of participants, we eventually refrained from doing so, because preliminary interviews revealed that the majority of the participants preferred a private atmosphere during the experimental session. Many of them even doubted to be able to be moved to tears when monitored by the experimenter or a camera. Moreover, we were also interested in subtle lacrimation states, i.e., feeling like crying, which is not observable neither by a present person nor by a camera. Importantly, however, the after-effects of crying remain visible in most cases in the face of the participant, as indicated by red sclera of the eyes and red areas around the eyes, wet cheeks, smeared make-up, running nose and presence of used tissues. When re-entering the experimental room, the experimenter checked first of all for these visual indicators. More than 90% of the sample showed positive evidence for a recent crying episode. We thus had no reason to be concerned about demand characteristics.

On the basis of converging evidence from this study, our earlier findings, and work reported by other groups, we claim that the display of prosocial behavior is particularly critical for the elicitation of feelings of being moved. This is especially true for art forms that are narrative in nature, such as novels, plays, films, songs, ballads, choreographic performances, and so forth (Schoeller and Perlovsky, 2015). However, the variety of stimuli capable of eliciting states of being moved also includes instrumental music, which does not display a social component as overtly as narrative genres do. Obviously, other processes must be at work to trigger these emotional responses, with musical expectancy representing one important factor (Huron and Margulis, 2010). It should also be noted that the evolutionary origin of instrumental music has been closely related to social activities and social emotions (Sloboda and O’Neill, 2001; Dissanayake, 2008; Panksepp, 2009). Thus, even instrumental music may activate latent associations with social cognition. In fact, music was also a predominant feature in our tears-eliciting stimulus set as compared to the control set. Since we did not manipulate any features of the film excerpts, in order to guarantee high ecological validity, we cannot quantify the share that music had in eliciting the states of being moved, compared to the displays of prosocial behavior. A neat disentanglement of these effects would require considerable additional effort, since original material with separated audio and video tracks would be needed to perform this kind of research. Similarly, we cannot quantify the share of other formal filmic features such as the predominant usage of short camera distances as compared to, for instance, the influence of emotional contagion through depiction of crying characters (which is significantly higher for tears-eliciting clips) or facial emotional expressions in general. Due to the correlational nature of our findings we cannot claim that the short camera distance or the eye-level perspective *produce* the tears response in the viewer. Rather, we think that these technical characteristics facilitate other effects: For example, in order to depict facial emotional expressions that are contagious for the viewer, both a short camera distance in regard to the face of the character and an eye-level perspective are almost mandatory. If the character’s face expressing a certain emotion is shot from a long distance or from a worm’s view, chances are low that this emotional expression will be detected by the viewer.

As mentioned in the introduction, scientific research on tears distinguishes five categories of emotional tears, with art-elicited tears representing one of these. Having studied emotional tears exclusively in response to film clips, we cannot generalize our findings to the other four categories of emotional tears. Since previous research has often conflated different types of emotional tears, we would like to emphasize once more that art-elicited tears may differ from other types of emotional tears because of their unique ability to be concomitant with an overall positively valenced emotional state of being moved.

## Conclusion

By identifying the psychophysiological correlates of art-elicited tears, their relationship to emotional piloerection, and the stimulus characteristics that facilitate these intense emotional responses, our study contributes to resolving some of the mysteries of “the secret land of tears” and promotes the understanding of a phenomenon that is unique to humans.

## Ethics Statement

All subjects gave written informed consent in accordance with the Declaration of Helsinki. All procedures were approved by the Ethics Council of the Max Planck Society. There were no vulnerable populations involved.

## Author Contributions

EW conceived, designed, and conducted the study, JH prepared the stimuli, EW performed the statistical analyses, MS coded the technical features of the clips, EW and WM wrote the manuscript, TJ revised the manuscript, all authors discussed the results and implications of the study.

## Conflict of Interest Statement

The authors declare that the research was conducted in the absence of any commercial or financial relationships that could be construed as a potential conflict of interest.

## Footnotes

1 Emotional tears are determined by the absence of a physical irritation of the eye (e.g., irritation from dust or molecules floating in the air).

2 The last category is embedded by Vingerhoets in the category of “sentimental tears.“ Due to the pejorative usage (Tan and Frijda, 1999; Solomon, 2004) of this term on the one hand, but a strong link to an overall positive emotion that we focus on here (being moved), we prefer to use “art-elicited” rather than “sentimental” (although the latter term may embrace a broader range of antecedents, i.e., not only art-related elicitors but also the beauty of nature, ceremonial acts such as weddings, etc.).

3 http://www.goosecam.de/

4 http://www.virtualdub.org/

5 http://www.ledalab.de/

6 Please note that we do not claim that the control set of matched clips was emotionally neutral for the participants. It is possible that due to the random nature of our extraction procedure some control clips could have evoked emotional responses in the participant. However, participants were instructed to focus on emotional peak points (usually one) within the films; moreover, if more than one such peak point had been indicated for a certain film, all of them were used in the study (cf. Table S4). Therefore, it is highly unlikely that the control set had the emotional power to elicit tears in the respective participants.

7 http://www.imdb.com/

8 Thus our data suggest that goosebumps are although strong, but not *peak* emotional responses, as unanimously assumed in the pertinent literature. Rather, the very peak levels of physiological arousal are exclusively reached by combinations of goosebumps and tears (goosetears), with a substantial gap between goosetears and goosebumps only. Because, to our knowledge, no study on piloerection and chills collected additional ratings regarding emotional lacrimation, one could speculate that in former studies goosebumps and goosetears were conflated. Critically, the presence of the latter category could have unknowingly amplified the physiological outcomes of the former one.

9 An alternative explanation of increased corrugator and zygomaticus activity might be that the process of crying can involve a great number of facial muscles, including the zygomaticus, without any expression of positive emotions. Although our interpretation of mixed-emotion states is backed by both previous research and verbal statements by our participants during the interview, we cannot definitely rule out this alternative interpretation.

10 The same mechanism of enhancement of (in-)group cohesion might likewise be at work in ritual weeping (Vingerhoets, 2013). Here, deliberately evoked collective crying is used in order to unify the members as a group in times of great difficulties or challenges (cf. Dissanayake, 2008).

11 For all these reasons, several studies on emotional tears have even limited their samples to female participants alone (e.g., Frey et al., 1981; Kraemer and Hastrup, 1988; Gross et al., 1994).
