# Supplementary material for: Tears Falling on Goosebumps: Co-occurrence of Emotional Lacrimation and Emotional Piloerection Indicates a Psychophysiological Climax in Emotional Arousal
Source: Front Psychol. 2017 Feb 7;8:41. doi: 10.3389/fpsyg.2017.00041 (PMC5293808; doi:10.3389/fpsyg.2017.00041)
Supplement: Supplementary file 1 [file Table_1.docx]

**Supplementary Material**

**
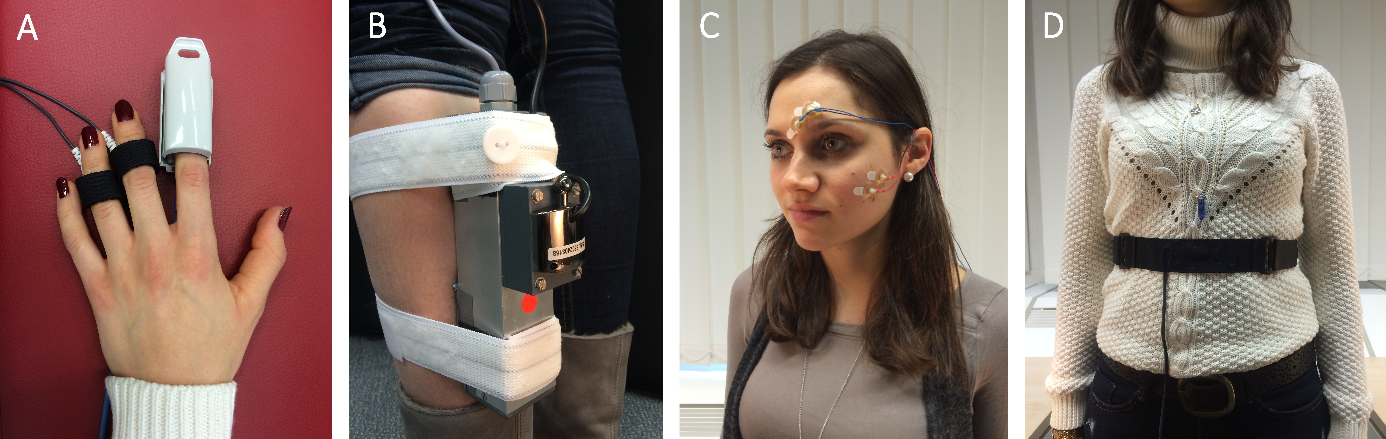
**

**Figure S1. Physiological measures. (A)** Measurement of electrodermal activity and blood volume pulse using two electrodes at the phalanges of the ring and middle fingers and a photoplethysmograph sensor on the index finger; **(B)** collection of piloerection data via a goosecam attached to the lower leg; **(C)**measurement of electromyographic activity over the corrugator supercilii above the eyebrow and zygomaticus major at the cheek; **(D)** collection of respiratory activity using a stretch-sensitive respiration belt placed around the diaphragm.

**
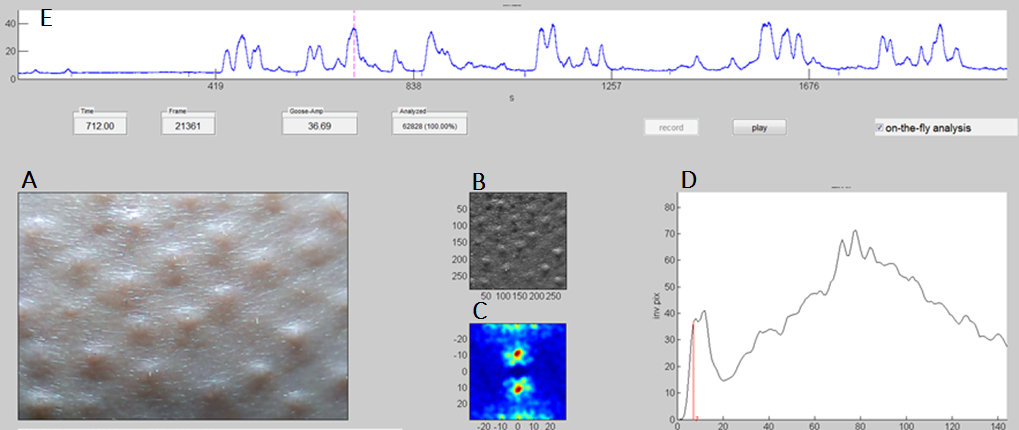
**

**Figure S2**. Screen shot of the analysis of a goosecam video, using the Matlab based software Gooselab V1.21. After a transformation of the original video frame **(A)** into a gray scale picture **(B)**, a two dimensional discrete Fourier transform **(C)** is performed, and converted to a one-dimensional spectrum of spatial frequency **(D)**. Using these data, a continuous measure of piloerection intensity **(E)** is derived for the whole experimental session (the flat curve at the beginning represents the baseline period). The dashed red curser in (E) is placed within a piloerection episode.


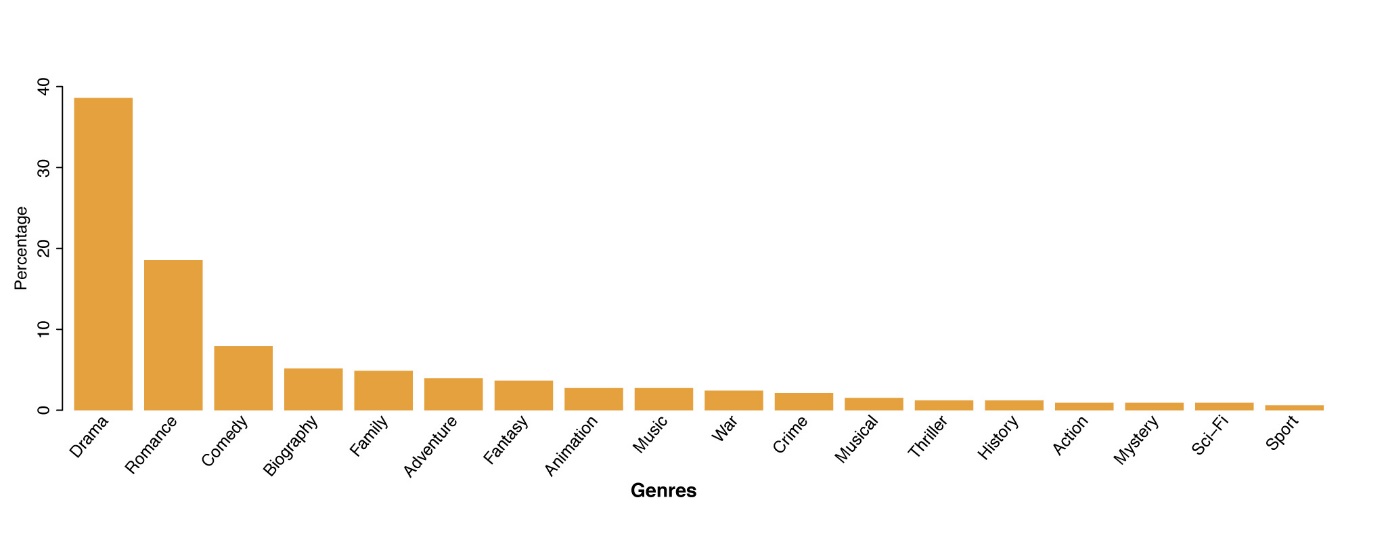


**Figure S3**. Distribution of the genres (according to the Internet Movie Data Base) from which the self-selected, tears-eliciting film clips were taken. Genres with a strong social tendency, such as the drama and romance genres, dominated the stimulus set. These genres are known to focus explicitly on societal values and virtues such as altruism, bonding, self-sacrifice, and faithfulness.


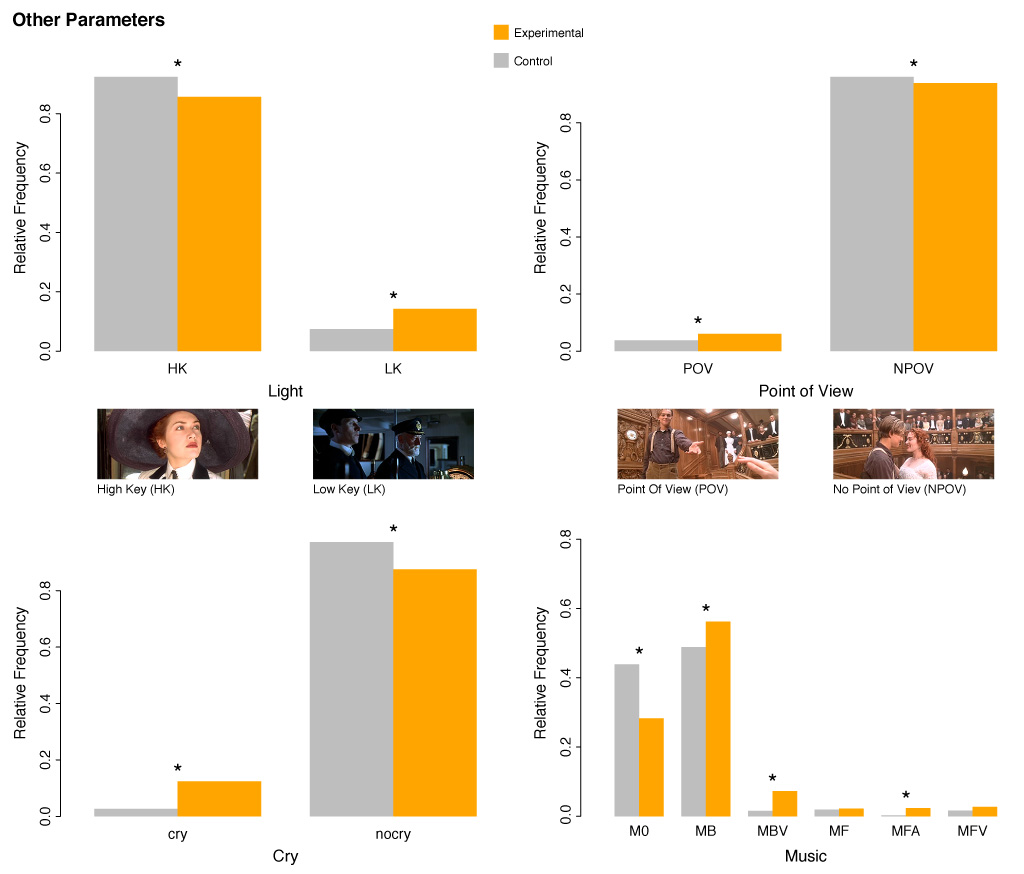


**Figure S4**. Comparison of lighting, point-of-view-shots, depiction of crying characters, and music between tears-eliciting clips and the matched control set. All groups differed significantly from each other in Pearson’s chi square tests (for details, see Tab. S5). For music, we differentiated between non-diegetic (i.e., outside of the story world) instrumental musical score (MB), non-diegetic vocal singing with instruments (MBV), diegetic music, i.e., depiction of music making within the film story world (MF), diegetic singing (MFA), and diegetic singing accompanied by instruments (MFV). Shots without any music were coded as M0. The pictures illustrating the lighting and the point-of-view shot were taken from *Titanic* (1997).

**Table S1**. Results of 2 × 2 mixed-effect analyses of variances for the psychophysiological signals.

|  | Fixed Effects | *df*_(num.den)_ | *F*-value | *p*-value |  |
| --- | --- | --- | --- | --- | --- |
| Phasic EDA | Intercept | 1, 64784 | 6.11 | 0.0134 | * |
|  | Tears | 1, 64784 | 422.33 | < .0001 | *** |
|  | Goose | 1, 64784 | 61.95 | < .0001 | *** |
|  | Tears × Goose | 1, 64784 | 29.24 | < .0001 | *** |
| Heart Rate | Intercept | 1, 66368 | 7.23 | 0.0071 | ** |
|  | Tears | 1, 66368 | 1005.59 | < .0001 | *** |
|  | Goose | 1, 66368 | 72.62 | < .0001 | *** |
|  | Tears × Goose | 1, 66368 | 9.30 | 0.0023 | ** |
| Corrugator | Intercept | 1, 66368 | 16.63 | < .0001 | *** |
|  | Tears | 1, 66368 | 3719.43 | < .0001 | *** |
|  | Goose | 1, 66368 | 28.83 | < .0001 | *** |
|  | Tears × Goose | 1, 66368 | 2.31 | 0.1285 |  |
| Zygomaticus | Intercept | 1, 64784 | 12.58 | < .001 | ** |
|  | Tears | 1, 64784 | 2137.78 | < .0001 | *** |
|  | Goose | 1, 64784 | 94.54 | < .0001 | *** |
|  | Tears × Goose | 1, 64784 | 54.21 | < .0001 | *** |
| Respiration Rate | Intercept | 1, 66368 | 15.10 | < .001 | ** |
|  | Tears | 1, 66368 | 78.17 | < .0001 | *** |
|  | Goose | 1, 66368 | 37.55 | < .0001 | *** |
|  | Tears × Goose | 1, 66368 | 41.50 | < .0001 | *** |

**Table S2**. Results of one-way mixed-effect analyses of variances for the psychophysiological signals with the binary factor Rating (feeling of tears vs. lacrimation).

|  | Fixed Effects | *df*_(num.den)_ | *F*-value | *p*-value |  |
| --- | --- | --- | --- | --- | --- |
| Phasic EDA | Intercept | 1, 17024 | 17.43 | < .0001 | *** |
|  | Rating | 1, 17024 | 346.24 | < .0001 | *** |
| Heart Rate | Intercept | 1, 17593 | 17.43 | < .0001 | *** |
|  | Rating | 1, 17593 | 346.24 | < .0001 | *** |
| Corrugator | Intercept | 1, 17593 | 20.38 | < .0001 | *** |
|  | Rating | 1, 17593 | 9.83 | 0.0017 | ** |
| Zygomaticus | Intercept | 1, 17593 | 13.25 | < .001 | ** |
|  | Rating | 1, 17593 | 330.60 | < .0001 | *** |
| Respiration Rate | Intercept | 1, 17593 | 9.46 | 0.0021 | ** |
|  | Rating | 1, 17593 | 85.58 | < .0001 | *** |

**Table S3**. Relative frequencies and standardized residuals from a significant Pearson’s chi square test (*χ²*= 126.64; *df* = 6; *p* < 0.001) for different camera distances of the tears-eliciting experimental and the matched control set. The last column indicates significant differences between the two sets; standardized residuals having absolute values greater than 3 indicate significant differences (Agresti, 2007, p.38^[[1]](#footnote-1)^).

| **Camera distance** | **Experimental set**  *(rel. frequencies, standardized residuals)* | | **Control Set**  *(rel. frequencies, standardized residuals)* | |  |
| --- | --- | --- | --- | --- | --- |
| Extreme close-up | 0.0144 | 1.7 | 0.0092 | -1.7 |  |
| Close-up | 0.130 | 8.0 | 0.064 | -8.0 | * |
| Medium close-up | 0.37 | 4.8 | 0.30 | -4.8 | * |
| Medium shot | 0.20 | -4.2 | 0.25 | 4.2 | * |
| Medium-long shot | 0.12 | -5.6 | 0.17 | 5.6 | * |
| Long shot | 0.16 | -3.1 | 0.19 | 3.1 | * |
| Extreme long shot | 0.0092 | 1.5 | 0.0056 | -1.5 |  |

**Table S4**. Relative frequencies and standardized residuals from a significant Pearson’s chi square test (*χ²*= 8.08; *df* = 4; *p* < 0.001) for different camera perspectives of the tears-eliciting and the control set. The last column indicates significant differences between the two sets; standardized residuals having absolute values greater than 3 indicate significant differences (Agresti, 2007, p.38).

| **Camera perspective** | **Experimental set**  *(rel. frequencies, standardized residuals)* | | **Control Set**  *(rel. frequencies, standardized residuals)* | |  |
| --- | --- | --- | --- | --- | --- |
| Worm’s view | 0.0074 | 1.1 | 0.0049 | -1.1 |  |
| Low angle | 0.051 | -6.9 | 0.103 | 6.9 | * |
| Eye-level | 0.82 | 5.9 | 0.75 | -5.9 | * |
| High angle | 0.099 | -3.1 | 0.127 | 3.1 | * |
| Bird’s view | 0.021 | 2.8 | 0.012 | -2.8 |  |

**Table S5**. Relative frequencies and standardized residuals from significant Pearson’s chi square tests for lighting (*χ²* = 61.73; *df* = 1; *p* < 0.001), point-of-view shots (*χ²* = 14.43; *df* = 1; *p* < 0.001), depiction of crying characters (*χ²* = 183.97; *df* = 1; *p* < 0.001), and music (*χ²* = 251.43; *df* = 5; *p* < 0.001; M0: no music, MB non-diegetic instrumental musical, MBV: non-diegetic singing accompanied by instruments, MF: diegetic music, MFA: diegetic singing, MFV: diegetic singing accompanied by instruments). The last column indicates significant differences between the two sets; standardized residuals having absolute values greater than 3 indicate significant differences (Agresti, 2007, p.38).

|  | **Experimental set**  *(rel. frequencies, standardized residuals)* | | **Control Set**  *(rel. frequencies, standardized residuals)* | |  |
| --- | --- | --- | --- | --- | --- |
| **Lighting** |  |  |  |  |  |
| high key | 0.86 | -7.9 | 0.92 | 7.9 | * |
| low key | 0.143 | 7.9 | 0.075 | -7.9 | * |
| **Point-of-view shots** |  |  |  |  |  |
| POV | 0.061 | 3.9 | 0.037 | -3.9 | * |
| no POV | 0.94 | -3.9 | 0.96 | 3.9 | * |
| **Depiction of crying** |  |  |  |  |  |
| crying | 0.124 | 14 | 0.026 | -14 | * |
| no crying | 0.88 | -14 | 0.97 | 14 | * |
| **Music** |  |  |  |  |  |
| M0 | 0.28 | -11.82 | 0.44 | 11.82 | * |
| MB | 0.56 | 4.95 | 0.49 | -4.95 | * |
| MBV | 0.072 | 10.14 | 0.016 | -10.14 | * |
| MF | 0.022 | 0.65 | 0.019 | -0.65 |  |
| MFA | 0.0236 | 6.88 | 0.0025 | -6.88 | * |
| MFV | 0.027 | 2.63 | 0.016 | -2.63 |  |

**Table S6**. Participants’ original wording describing the subjective experience of being moved to tears. The “Valence” column shows the overall tendency of participants to label these episodes as rather pleasant, unpleasant, or mixed. Some participants could not (or did not want to) give an answer (–).

| **Subj** | **Valence** | **English Translation** | **Original Description** |
| --- | --- | --- | --- |
| #14 | – | – | – |
| #15 | pleasant | related to nice memories | verbunden mit schönen Erinnerungen |
| #21 | mixed | not unpleasant, but not pleasant either, right in the middle of these | nicht unangenehm, aber auch nicht richtig angenehm, mitten drin |
| #34 | – | – | – |
| #44 | unpleasant | I like to watch these films repeatedly, because there are also other moments | ich schaue alle Filme gerne mehrmals, weil da auch andere Momente sind |
| #45 | pleasant | liberating; one likes to see it, because the scene is emotional and touching | befreiend; man sieht es gerne, weil die Szene emotional und berührend ist |
| #46 | pleasant | although unpleasant themes, but “true”; good for psychic hygiene | zwar unangenehme Themen, aber „wahr“; gut für seelische Hygiene |
| #47 | – | – | – |
| #48 | mixed | during the experiment rather unpleasant, but at home rather pleasant | im Experiment eher unangenehm, aber daheim eher angenehm |
| #49 | pleasant | beautiful; so sad; warmth is bottled up in the chest, the only way out is through the eyes; liberating | schön; so traurig; Anstauen von Wärme in der Brust, der einzige Weg raus ist durch die Augen; befreiend |
| #51 | pleasant | it feels good to release it; beautiful, because one sympathizes; one is engaged, not distanced; important things in life are shifted to the foreground (obligations and stress are forgotten); one is more by oneself; relaxing; not stressful; one catches one’s breath and sighs; when the tears are over, one is present | rauslassen tut gut; schön, weil man mitfühlt; man lässt sich ein, ist nicht distanziert; wichtige Dinge im Leben werden in den Vordergrund gerückt (Termine und Stress werden vergessen); man ist mehr bei sich; entspannend; nicht stressend; man atmet durch und seufzt; wenn Tränen zu Ende gehen, ist man präsent |
| #56 | mixed | it depends on the mood; sometimes pleasant and liberating; I’m becoming obsessed about it, need time to distance myself from it mentally; it heals only later | hängt von Stimmung ab; manchmal angenehm und befreiend; ich steigere mich rein, brauche einige Zeit, um mich gedanklich wieder davon zu distanzieren; es heilt nach |
| #71 | unpleasant | unpleasant, especially when others are around and see you cry; alone it’s okay | unangenehm, v.a. auch wenn andere dabei sind und dich weinen sehen, alleine okay |
| #77 | pleasant | liberating, overwhelming | befreiend, überwältigend |
| #78 | pleasant | beautiful tears, but unpleasant portions resonate, too | schöne Tränen, aber auch unangenehme Anteile schwingen mit |
| #79 | mixed | beautifully sad | schön traurig |
| #80 | mixed | inner pressure is built up and wants to get out at the top; locked feelings; the feeling of tears is a relief | innerlicher Druck, der sich aufbaut und nach oben raus will; verschlossene Gefühle; Tränengefühl ist eine Erleichterung |
| #81 | mixed | physically exhausting; I’d like to flush away the lump in the throat with a glass of water; beautiful stories, though sad | körperlich anstrengend; ich möchte am liebsten das Kloßgefühl wegspülen mit einem Glas Wasser; schöne Geschichten auch wenn traurig |
| #82 | pleasant | liberating | befreiend |
| #84 | pleasant | one cries for joy, too; here it is also a beautiful feeling; difficult to describe; when one cries, it somehow makes you happy, sort of a liberating feeling | man weint ja auch vor Glück; hier ist es auch ein schönes Gefühl; schwer zu beschreiben; wenn man weint, macht es mich danach irgendwie glücklich, so ein befreiendes Gefühl |
| #85 | pleasant | good feeling, tingling; you don’t try to avoid it; you sympathize and that’s good; you think, you don’t want to have this experience yourself | gutes Gefühl, Kribbeln; man geht dem nicht aus dem Weg; man fühlt mit und das ist gut; man denkt, man will es selber nicht erleben |
| #86 | – |  | – |
| #87 | – |  | – |
| #88 | pleasant | constricting feeling at the beginning; if one cries, it is liberating, if not, the constricting feeling remains; one feels more alive; intense feeling; the tear-jerking scenes are, however, not my favorite ones | beklemmendes Gefühl am Anfang; wenn man weint, ist es dann befreiend, wenn nicht, bleibt das beklemmendes Gefühl bestehen; man fühlt sich lebendiger; intensives Gefühl; die Heulszenen sind aber nicht meine Lieblingsszenen |
| #90 | mixed | I do like to be touched by scenes; like to watch such movies; that constitutes a good movie | ich mag schon, wenn mich die Szenen berührt; schaue gerne solche Filme; das macht einen guten Film aus |
|  |  |  |  |

**Table S7**. List of all tears-eliciting clips self-selected by participants. The “Count” column indicates the number of participants who selected the clip. The “Genre” column is based on the classification by the Internet Movie Data Base (IMDb). The “Emotion” column identifies the predominant emotion of the scenario type (sadness or joy).

| **Clip** | **Film Title** | **Year** | **Country** | **Length** | **Count** | **Genre** | **Emotion** | **Scenario** |
| --- | --- | --- | --- | --- | --- | --- | --- | --- |
|  |  |  |  |  |  |  |  |  |
| 1 | Gladiator | 2000 | USA | 03:04 | 1 | Action, Drama | sadness | death of wife and son |
| 2 | Lilo and Stich | 2002 | USA | 03:34 | 1 | Animation, Adventure, Comedy | sadness | being lost in a strange world |
| 3 | Star Wars: Episode I | 1999 | USA | 04:22 | 1 | Action, Adventure, Fantasy | sadness | leaving enslaved mother behind to become a Jedi |
| 4 | Up | 2009 | USA | 07:45 | 2 | Animation, Adventure, Comedy | sadness | wife dies before the fulfillment of her lifetime dream |
| 5 | Wall-E | 2008 | USA | 02:06 | 1 | Animation, Adventure, Family | joy | realizing that one’s own hostile reaction was unjustified; reconciliation |
| 6 | Wall-E | 2008 | USA | 05:13 | 1 | Animation, Adventure, Family | joy | caring for love interest who is in a state of hibernation |
| 7 | Beaches | 1988 | USA | 04:16 | 1 | Comedy, Drama, Music | sadness | death of mother; farewell |
| 9 | My Sister’s Keeper | 2009 | USA | 05:02 | 1 | Drama | sadness | terminally ill girl consoles her mother, says farewell and dies |
| 10 | In Her Shoes | 2005 | USA, Germany, UK | 02:30 | 1 | Comedy, Drama, Romance | joy | two sisters reconcile on one sister’s wedding day |
| 11 | The Other Woman | 2009 | USA | 03:31 | 1 | Drama | sadness | woman rejected by her husband after her infant dies |
| 12 | The Other Woman | 2009 | USA | 04:35 | 1 | Drama | joy | female main character finally bonds with her stepson |
| 13 | Hachi: A Dog’s Tale | 2009 | USA, UK | 04:25 | 1 | Drama, Family | sadness | memories from dog’s perspective; dog dies |
| 14 | Hachi: A Dog’s Tale | 2009 | USA, UK | 02:01 | 2 | Drama, Family | sadness | widow discovers her deceased husband’s dog waiting for him at the train station |
| 15 | The Notebook | 2004 | USA | 04:10 | 1 | Drama, Romance | joy | couple on a boat kiss passionately in the rain |
| 16 | The Notebook | 2004 | USA | 05:35 | 1 | Drama, Romance | sadness | woman reads letters of regret and cries |
| 17 | The Notebook | 2004 | USA | 04:37 | 1 | Drama, Romance | sadness | woman with dementia has a breakdown, forgets that the man in front of her is her former lover |
| 18 | The Notebook | 2004 | USA | 05:25 | 2 | Drama, Romance | sadness | couple in nursing home passing away after announcing their mutual love |
| 19 | One Day | 2011 | USA, UK | 05:30 | 1 | Drama, Romance | sadness | woman dies on the way home to her partner |
| 20 | Titanic | 1997 | USA | 04:07 | 3 | Drama, Romance | sadness | male protagonist frozen to death; woman being rescued against all odds |
| 21 | Troy | 2004 | USA, Malta, UK | 06:42 | 1 | Adventure | joy | father finally convinces Achilles to return the bones of his dead son |
| 22 | Darjeeling Limited | 2007 | USA | 02:46 | 1 | Adventure, Comedy, Drama | sadness | men try to save drowning children; one child dies; they carry his body to his family |
| 23 | Friends | 1994–2004 | USA | 02:35 | 1 | Comedy, Romance | sadness | couple breaks up |
| 24 | Marley & Me | 2008 | USA | 04:16 | 3 | Comedy, Drama, Romance | sadness | dog is being euthanized; owner says goodbye |
| 25 | Mar adentro | 2004 | Spain, France, Italy | 04:16 | 1 | Biography, Drama, Romance | sadness | paraplegic commits suicide after losing a battle for the legalization of euthanasia |
| 26 | Mary Poppins | 1964 | USA | 03:44 | 1 | Comedy, Family, Fantasy | joy | babysitter sings a lullaby |
| 28 | Chrono Crusade | 2003–2004 | Japan | 05:15 | 1 | Animation, Adventure, Drama | sadness | male and female protagonists die peacefully together |
| 29 | Grey‘s Anatomy | 2005– | USA | 02:09 | 1 | Drama, Romance | sadness | woman getting divorced and forced to change her career plan |
| 30 | Grey‘s Anatomy | 2005– | USA | 04:45 | 1 | Drama, Romance | sadness | two medical coworkers die, one of whom is the girlfriend of the doctor |
| 31 | My Girl | 1991 | USA | 04:32 | 2 | Comedy, Drama, Family | sadness | funeral of girl’s only friend; daughter can’t accept his death; father unable to console her |
| 32 | A Single Man | 2009 | USA | 03:49 | 1 | Drama | sadness | man has heart failure while feeling happy for the first time in a long while |
| 33 | Citizen Kane | 1941 | USA | 03:03 | 1 | Drama, Mystery | sadness | what remains from a man’s childhood is burned in a fire |
| 34 | Paths of Glory | 1957 | USA | 04:49 | 1 | Drama, War | sadness | female prisoner of war made to sing in front of soldiers |
| 35 | Vertigo | 1958 | USA | 02:26 | 1 | Mystery, Romance, Thriller | joy | man kisses love interest who is thought to be dead |
| 36 | 21 Grams | 2003 | USA | 02:26 | 1 | Crime, Drama, Thriller | sadness | man shoots himself; dies in hospital after internal monologue |
| 37 | Léon | 1994 | France | 07:16 | 1 | Crime, Drama, Thriller | sadness | a girl’s mentor is killed; she grieves for him |
| 38 | Precious | 2009 | USA | 05:44 | 1 | Drama | sadness | mother relates how she and her daughter were abused by her partner |
| 39 | Requiem for a Dream | 2000 | USA | 03:55 | 1 | Drama | sadness | conclusion of the storylines of several drug addicts |
| 40 | Almanya | 2011 | Germany | 03:21 | 1 | Comedy, Drama | sadness | family road trip; father dies suddenly |
| 41 | Kirschblüten | 2008 | Germany | 05:26 | 1 | Drama, Romance | sadness | mother dies; son regrets not having spent enough time with her |
| 42 | Kirschblüten | 2008 | Germany | 04:48 | 1 | Drama, Romance | sadness | man dies; reunited with his wife in death |
| 44 | Der Untergang | 2004 | Germany, Austria, Italy | 05:29 | 1 | Biography, Drama, War | joy | end of WW II; female German soldier disarmed and left alive |
| 45 | Seven Pounds | 2008 | USA | 02:34 | 1 | Drama, Romance | joy | organ recipients with common donor (who committed suicide for them) meet for the first time |
| 46 | Hachi: A Dog’s Tale | 2009 | USA, UK | 03:54 | 1 | Drama, Family | sadness | dog waits for his deceased owner at the usual spot |
| 48 | Les Choristes | 2004 | France, Switzerland, Germany | 01:29 | 1 | Drama, Music | joy | children find a way to circumvent their detention to say goodbye to a beloved teacher |
| 49 | My Sister’s Keeper | 2009 | USA | 02:25 | 1 | Drama | sadness | during a court session, the brother of terminally ill sister reveals her wish to die |
| 50 | My Sister’s Keeper | 2009 | USA | 02:25 | 1 | Drama | sadness | terminally ill child apologizes to her family for being a burden |
| 51 | Message in a Bottle | 1999 | USA | 01:28 | 1 | Drama, Romance | sadness | woman throws bottle with a message into the ocean |
| 52 | The Pursuit of Happiness | 2006 | USA | 04:02 | 2 | Biography, Drama | sadness | homeless father and son forced to spend the night in a public restroom |
| 53 | Dragonfly | 2002 | USA, Germany | 04:03 | 1 | Drama, Fantasy, Mystery | joy | widower visits an African tribe and finds that his wife gave birth to a girl before she died |
| 54 | My Sister’s Keeper | 2009 | USA | 03:37 | 1 | Drama | joy | family with terminally ill daughter has a nice day at the beach |
| 55 | Hilde | 2009 | Germany | 02:23 | 1 | Biography, Drama, Music | joy | woman discovers her passion for singing |
| 56 | Hilde | 2009 | Germany | 04:30 | 1 | Biography, Drama, Music | joy | woman is praised for her singing talent |
| 57 | La vita è bella | 1997 | Italy | 03:01 | 1 | Comedy, Drama, Romance | sadness | father and son board a train to a concentration camp; father hides the truth with humorous lies |
| 58 | La vita è bella | 1997 | Italy | 01:17 | 1 | Comedy, Drama, Romance | joy | woman awakened by the music her husband manages to play in the concentration camp |
| 59 | La vita è bella | 1997 | Italy | 02:44 | 2 | Comedy, Drama, Romance | joy | boy is freed from a concentration camp and reunited with his mother |
| 60 | Marie Antoinette | 2006 | USA, France, Japan | 01:40 | 1 | Biography, Drama, History | sadness | woman is bored by a social event and wants to leave; daydreams of an officer in battle |
| 62 | Rabbit Hole | 2010 | USA | 03:09 | 1 | Drama | sadness | husband accuses wife of trying to erase all memories of their deceased son’s existence |
| 65 | I Am Sam | 2001 | USA | 01:06 | 1 | Drama | sadness | father and daughter are being separated |
| 66 | I Am Sam | 2001 | USA | 02:53 | 1 | Drama | joy | foster mother returns child to mentally handicapped father |
| 67 | I Am Sam | 2001 | USA | 02:20 | 1 | Drama | joy | daughter lies to the court in order to stay with her mentally handicapped father |
| 69 | Benjamin Button | 2008 | USA | 05:08 | 1 | Drama, Fantasy, Romance | sadness | protagonist dies in the arms of his beloved |
| 70 | Ghost | 1990 | USA | 03:39 | 1 | Drama, Fantasy, Romance | joy | passionate scene between two lovers |
| 71 | Ghost | 1990 | USA | 04:26 | 1 | Drama, Fantasy, Romance | joy | ghost of a deceased man takes control of a medium’s body to dance with his wife |
| 72 | Ghost | 1990 | USA | 03:30 | 1 | Drama, Fantasy, Romance | joy | woman sees her deceased husband’s ghost one last time before he vanishes |
| 73 | The Lion King | 1994 | USA | 05:20 | 1 | Animation, Adventure, Drama | sadness | Simba tries to wake up his dead father and realizes it’s not going to happen |
| 74 | The Horse Whisperer | 1998 | USA | 06:22 | 1 | Drama, Romance, Western | sadness | two girls have an accident while riding their horses; one girl dies |
| 75 | Beginners | 2010 | USA | 03:54 | 1 | Comedy, Drama, Romance | sadness | couple breaks up |
| 76 | Beginners | 2010 | USA | 02:12 | 1 | Comedy, Drama, Romance | sadness | father dies peacefully at home with family and friends around him |
| 77 | Big Fish | 2003 | USA | 11:06 | 1 | Adventure, Drama, Fantasy | sadness | father dies after son tells him a reassuring fantasy version of how all his friends say goodbye |
| 78 | Love Actually | 2003 | UK, USA, France | 06:52 | 1 | Comedy, Drama, Romance | joy | father encourages boy to break through airport security to see his love interest before she flies away |
| 79 | Love Actually | 2003 | UK, USA, France | 02:17 | 1 | Comedy, Drama, Romance | joy | man confesses his love to a good friend who is about to marry someone else |
| 80 | Finding Neverland | 2004 | USA, UK | 05:45 | 1 | Biography, Drama, Family | joy | children arrange a moving theater play for their sick mother |
| 81 | Dumbo | 1941 | USA | 02:51 | 1 | Animation, Family, Musical | joy | little elephant is briefly reunited with his imprisoned mother |
| 82 | Komt een vrouw bij de dokter | 2009 | Netherlands | 04:28 | 1 | Drama, Romance | sadness | terminally ill woman has a breakdown; says goodbye to husband and child |
| 83 | Les Misérables | 2012 | USA, UK | 02:59 | 1 | Drama, Musical, Romance | sadness | ill beggar on the deathbed; has hallucinations of her daughter |
| 84 | My Sister’s Keeper | 2009 | USA | 03:48 | 1 | Drama | sadness | terminally ill girl asks her sister to help her resist their mother’s intention to treat her illnesses further |
| 85 | Soul Surfer | 2011 | USA | 03:24 | 1 | Biography, Drama, Sport | sadness | daughter sees the stump where her arm should be for the first time after a shark attacked her |
| 86 | The Last Unicorn | 1982 | USA, UK, Japan, Germany | 03:21 | 1 | Family, Animation, Fantasy | joy | unicorn resurrects dead person, then leaves him |
| 87 | E.T. the Extra-Terrestrial | 1982 | USA | 04:10 | 2 | Family, Sci-Fi | sadness | alien leaves planet earth after saying goodbye to a boy who befriended it |
| 88 | Sweet November | 2001 | USA | 05:37 | 1 | Drama, Romance | sadness | woman leaves the only man she ever loved because she has cancer and doesn’t want him to see her die |
| 89 | Lord of the Rings: III | 2003 | USA, New Zealand | 07:28 | 1 | Adventure, Drama, Fantasy | sadness | Bilbo, Frodo, and Gandalf leave middle-earth |
| 90 | Barfuss | 2005 | Germany | 04:08 | 1 | Comedy, Drama, Romance | sadness | girl contemplates suicide because she is unable to get in touch with her love interest |
| 91 | The Object of My Affection | 1998 | USA | 04:36 | 1 | Comedy, Drama, Romance | sadness | woman falls in love with her friend; he is gay and does not have the same feelings for her |
| 93 | The Pursuit of Happiness | 2006 | USA | 03:01 | 2 | Biography, Drama | joy | man finally gets the job he desperately needed |
| 94 | Artificial Intelligence: AI | 2001 | USA | 08:07 | 1 | Adventure, Drama, Sci-Fi | sadness | android boy nearly killed another (human) boy; his “mother” leaves him behind in the woods |
| 95 | Intouchables | 2011 | France | 05:56 | 1 | Biography, Comedy, Drama | joy | caregiver cheers up a depressed quadriplegic; arranges a date with the woman he is fond of |
| 96 | Precious | 2009 | USA | 03:28 | 1 | Drama | sadness | girl breaks down in class after she is told she contracted HIV from her father (who raped her) |
| 98 | The Ides of March | 2011 | USA | 01:20 | 1 | Drama | sadness | father gives eulogy at his daughter's funeral |
| 99 | The Ides of March | 2011 | USA | 02:18 | 1 | Drama | sadness | man finds his love interest after she commits suicide |
| 100 | Match Point | 2005 | UK, Russia, Ireland, USA | 04:47 | 1 | Drama, Romance, Thriller | sadness | man shoots his lover who refuses to abort her pregnancy and cover up the affair |
| 101 | Pride and Prejudice | 1995 | UK | 06:17 | 1 | Drama, Romance | sadness | man confesses his love to a woman from a rival family and gets harshly rejected |
| 102 | Doctor Zhivago | 1965 | USA, Italy, UK | 06:07 | 1 | Drama, Romance, War | sadness | man finds his beloved in the streets, follows her but dies before he reaches her |
| 103 | Blue Valentine | 2010 | USA | 03:18 | 1 | Drama, Romance | sadness | montage of a couple marrying and breaking up |
| 104 | Blue Valentine | 2010 | USA | 09:15 | 1 | Drama, Romance | sadness | relationship between a married couple deteriorates |
| 105 | Voshoschdenje | 1977 | Soviet Union | 04:24 | 1 | Drama, War | sadness | soldier tries to commit suicide after he commits treason |
| 106 | Ivanovo detstvo | 1962 | Soviet Union | 02:52 | 1 | Drama, War | joy | two characters are reunited during war time |
| 107 | Sudba cheloveka | 1959 | Soviet Union | 03:26 | 1 | Drama, War | joy | orphaned child reunited with his father who supposedly died during the war |
| 108 | Dancer in the Dark | 2000 | Denmark et al. | 06:23 | 2 | Crime, Drama, Musical | sadness | woman is being hanged; finds solace in the fact that her sacrifice was not in vain |
| 109 | Marvin’s Room | 1996 | USA | 06:01 | 1 | Drama | joy | two sisters bond after not having much contact over the last few years |
| 110 | The English Patient | 1996 | USA, UK | 05:32 | 1 | Drama, Romance, War | sadness | doctor has to leave his love interest behind to get help |
| 111 | P.S. I Love You | 2007 | USA | 00:53 | 1 | Drama, Romance | joy | widow finds a letter from her deceased husband, helping her to move on with her life |
| 112 | Schindler’s List | 1993 | USA | 03:17 | 1 | Biography, Drama, History | joy | Schindler gives water to people crammed into the trains to the concentration camp on a hot day |
| 113 | Sophie’s Choice | 1982 | UK, USA, France | 05:35 | 1 | Drama, Romance | sadness | Nazi officer forces Jewish woman to choose which of her children will have to die |
| 115 | Dancer in the Dark | 2000 | Denmark and others | 02:34 | 1 | Crime, Drama, Musical | sadness | woman gets fired due to her loss of eyesight |
| 116 | Emergency Room | 1994–2009 | USA | 01:31 | 1 | Drama | joy | grandmother reunited with her lost grandchild |
| 117 | Hachi: A Dog’s Tale | 2009 | USA, UK | 03:29 | 1 | Drama, Family | sadness | funeral of a man; his dog is shown going to the train station and waiting for his owner |
| 118 | Aimée & Jaguar | 1999 | Germany | 04:33 | 1 | Biography, Drama, Romance | sadness | two women are in love; one of them is imprisoned by the Nazis because of her Jewish ancestry |
| 119 | The Green Mile | 1999 | USA | 05:38 | 1 | Crime, Drama, Fantasy | joy | wrongly imprisoned death-row prisoner revives pet of other inmate |
| 120 | The Green Mile | 1999 | USA | 04:05 | 1 | Crime, Drama, Fantasy | sadness | last conversation between a prison guard and a befriended death row inmate before the execution |
| 121 | Philadelphia | 1993 | USA | 04:55 | 1 | Drama | sadness | patient with AIDS tells his boyfriend that he is ready to die |
| 122 | Schindler’s List | 1993 | USA | 04:30 | 1 | Biography, Drama, History | sadness | inmates of a concentration camp are forced to burn the corpses of other inmates |
| 123 | Schindler’s List | 1993 | USA | 02:50 | 1 | Biography, Drama, History | joy | women are pushed into the shower room in a concentration camp; the showers are real; they are relieved |
| 125 | Brokeback Mountain | 2005 | USA, Canada | 03:38 | 1 | Drama, Romance | sadness | man taking clothes of his dead lover as a reminder of their relationship |
| 126 | P.S. I Love You | 2007 | USA | 02:13 | 1 | Drama, Romance | joy | widow receives the last letter her husband wrote |
| 127 | P.S. I Love You | 2007 | USA | 02:54 | 1 | Drama, Romance | joy | widow reads letter from dead husband; it describes their first chance meeting and first kiss |
| 128 | P.S. I Love You | 2007 | USA | 02:27 | 1 | Drama, Romance | joy | widow receives tape recorder on her birthday; husband recorded a message before his death |
| 129 | P.S. I Love You | 2007 | USA | 01:48 | 1 | Drama, Romance | sadness | widow brings the urn of her dead husband home |
| 130 | P.S. I Love You | 2007 | USA | 01:43 | 1 | Drama, Romance | sadness | widow talks to her mother and cries |
| 131 | Titanic | 1997 | USA | 03:48 | 1 | Drama, Romance | sadness | woman dies; is reunited with her love interest in the “afterlife’ |
| 132 | Pearl Harbor | 2001 | USA | 06:31 | 1 | Action, Drama, Romance | sadness | soldier dies; on his deathbed he is told his wife expects a child |
| 133 | Napola | 2004 | Germany | 05:17 | 1 | Drama, Sport, War | sadness | young athletes forced to dive into a frozen lake; one of them commits suicide; his friend cannot save him |
| 135 | Desert Flower | 2009 | UK, Germany, France | 05:21 | 1 | Biography, Drama | sadness | woman recalls how she had her genitals mutilated as a child |
| 136 | Das weinende Kamel | 2003 | Germany, Mongolia | 06:24 | 1 | Documentary, Drama, Family | joy | camel finally accepted by its mother through a musical ceremony |
| 137 | Liebe in Gedanken | 2004 | Germany | 02:17 | 1 | Drama, Romance | sadness | love triangle; one of the three feels rejected and leaves |

1. Agresti, A. (2007). An Introduction to Categorical Data Analysis, 2nd Edn. Hoboken, NJ: JohnWiley & Sons. [↑](#footnote-ref-1)
